# Comparison of laser Doppler imaging, fingertip lacticemy test, and nailfold capillaroscopy for assessment of digital microcirculation in systemic sclerosis

**DOI:** 10.1186/ar3112

**Published:** 2010-08-10

**Authors:** Marcelo JU Correa, Luis EC Andrade, Cristiane Kayser

**Affiliations:** 1 Rheumatology Division, Department of Medicine, Universidade Federal de São Paulo, Rua Botucatu 740 3° andar, São Paulo 04023-062, Brazil

## Abstract

**Introduction:**

Laser Doppler imaging (LDI) is a relatively new method for assessing the functional aspect of superficial skin blood flow in systemic sclerosis (SSc) and Raynaud's phenomenon. The present study investigated the dynamic behavior of digital skin microvascular blood flow before and after cold stimulus (CS) in SSc patients and in healthy controls by means of a comprehensive approach of the functional (LDI), morphological (nailfold capillaroscopy (NFC)), and biochemical (fingertip lacticemy (FTL)) microcirculation components.

**Methods:**

Forty-four SSc patients and 40 healthy controls were included. After acclimatization, all subjects underwent NFC followed by LDI and FTL measurement. NFC was performed with a stereomicroscope under 10× to 20× magnification in the 10 digits of the hands. Skin blood flow of the dorsum of four fingertips (excluding the thumb) of the left hand was measured using LDI at baseline and for 30 minutes after CS. The mean finger blood flow (FBF) of the four fingertips was expressed as arbitrary perfusion units. FTL was determined on the fourth left finger before (pre-CS-FTL) and 10 minutes after CS.

**Results:**

LDI showed significantly lower mean baseline FBF in SSc patients as compared with controls (296.9 ± 208.8 vs. 503.6 ± 146.4 perfusion units; *P *< 0.001) and also at all time points after CS (*P *< 0.001). There was a significant decrease in mean FBF after CS as compared with baseline in SSc patients and in controls, followed by recovery of the blood flow 27 minutes after CS in healthy controls, but not in SSc patients. FBF tended to be lower in patients with digital scars and previous ulceration/amputation (*P *= 0.06). There was no correlation between mean baseline FBF and NFC parameters. Interestingly, there was a negative correlation between FTL and FBF measured by LDI in basal conditions and 10 minutes after CS in SSc patients.

**Conclusions:**

LDI showed lower digital blood flow in SSc patients when compared with healthy controls and correlated well with FTL both at baseline and after CS, allowing objective measurement of blood perfusion in SSc patients. The lack of correlation between functional and morphological microvascular abnormalities, measured by LDI and NFC, suggests they are complementary tools for evaluation of independent microangiopathy aspects in SSc patients.

## Introduction

Raynaud's phenomenon (RP) is a relatively common disorder characterized by episodic vasospasm of the extremities with a triphasic color change (blanching, cyanosis, and erythema) that usually occurs after exposure to cold or emotional stress [1]. The disorder can be idiopathic (primary RP) or secondary to systemic or local conditions. Secondary RP is a frequent finding in autoimmune rheumatic diseases, especially in systemic sclerosis (SSc), and is often more severe than primary RP [2]. In patients with SSc, structural abnormalities of the vasculature involving the microcirculation and the digital arteries, dysfunctional control of vascular tone, endothelial activation/lesion, and increased platelet adhesion can be detected early [3,4]. These alterations may result in progressive reduction of vessel lumen, decreased blood flow, and a state of chronic hypoxia, resulting in digital ulcers, digital pitting, and - in more severe cases - gangrene of the extremities.

Several methods have been developed to assess microvascular structural and functional abnormalities in patients with SSc in order to study the mechanisms of RP attacks, for differential diagnosis between primary RP and secondary RP, and for measuring responsiveness to treatment [5].

SSc peripheral microangiopathy can be easily recognized by widefield nailfold capillaroscopy (NFC), a noninvasive and safe method that is well established in the investigation of patients with RP [6-8]. In healthy individuals, the NFC pattern is characterized by homogeneous distribution of capillary loops similar in size and shape [7,8]. Some variability can be observed but avascular fields and megacapillaries are usually not detected [9]. Conversely, patients with SSc exhibit a typical NFC pattern characterized by enlargement of capillary loops, loss of capillaries, a variable degree of microhemorrhage, disruption of the orderly appearance of the capillary bed, and distortion of capillaries [8,10].

Lactate analysis is a common procedure in exercise physiology laboratories to measure endurance performance [11,12]. Determination of the lactic acid concentration in blood obtained from fingertips (fingertip lacticemy (FTL)) before and after a cold stimulus (CS) has been previously developed by our group for assessment of the biochemical component of peripheral perfusion, and has been shown to provide accurate information on the degree of local anerobiosis in patients with RP and SSc [13,14]. In brief, a drop of fingertip blood for lactic acid determination is obtained in resting conditions (pre-CS-FTL) and after a CS (post-CS-FTL). Our previous results showed higher pre-CS-FTL and post-CS-FTL levels in SSc patients as compared with normal controls and primary RP patients, reflecting the high degree of tissue hypoxia before and after a cold challenge in SSc patients [13-15].

The relatively new technique of laser Doppler imaging (LDI) allows an objective measurement of superficial cutaneous microvascular blood flow, and constitutes a promising approach in the assessment of the digital microvascular vasoreactivity in response to CS in SSc [16,17]. LDI has the advantages of being non-invasive and of mapping tissue perfusion over a wide area of the skin, thus providing more reproducible values [18]. The principle of the LDI technique is based on the Doppler effect, where changes in wavelength due to movement between red blood cells and the observer (the scanner) are used to determine the speed of blood flow [16]. Images are obtained by scanning a low-energy laser beam across the tissue. Backscattered light from the tissue, incident on a detector, is processed to provide a signal that is directly proportional to the speed and concentration of moving red blood cells [16,19]. Although promising, only few studies evaluated LDI associated with vasoreactive tests in patients with RP and SSc [16,17,20].

The aims of the present study were to evaluate the dynamic behavior of digital skin microvascular blood flow before and after CS using LDI in patients with RP secondary to SSc compared with healthy controls, and to correlate digital skin microvascular blood flow (measured by LDI) with structural microvascular abnormalities (evaluated by NFC) and with biochemical peripheral abnormalities (evaluated by the CS-FTL test) in patients with SSc.

## Materials and methods

### Patients

Forty-four patients with RP secondary to SSc meeting the American College of Rheumatology criteria [21] or the LeRoy and Medger criteria for early SSc [22] were consecutively selected from the Scleroderma Outpatient Clinic at UNIFESP Medical School Hospital. Forty healthy age-matched and gender-matched controls were selected among students and hospital employees at the same institution.

Exclusion criteria were the existence of active fingertip ulceration, smoking, occupational exposure to a cold environment and to vibratory agents, uncontrolled systemic arterial hypertension or diabetes mellitus, and clinical evidence of proximal arterial disease. Patients stopped oral vasodilators or any other medication for RP 3 days prior to the procedure. All individuals filled out an informed consent form and underwent a thorough rheumatologic examination. The study was approved by the UNIFESP Ethics Committee (reference number 1298/06).

### Study design

After acclimatization for 60 minutes in a laboratory with a constant temperature of 24 ± 1°C, all individuals underwent the following procedures: NFC, measurement of finger skin blood flow with the LDI, and measurement of FTL. The LDI and FTL measurements were performed before and after a CS.

### Nailfold capillaroscopy

All NFC procedures were performed in a stereomicroscope (SZ40; Olympus, Melville, NY, USA) under 10× to 20× magnification according to the protocol proposed by Andrade and colleagues [7]. A transparent ruler is incorporated into the right eyepiece of the stereomicroscope, allowing reproducible measurements of capillary width and of the number of capillary loops per millimeter. All 10 digits of the hands were examined except when prevented by extremely poor visibility or amputation.

The following parameters were analyzed: the number of capillary loops per millimeter, the vascular deletion score, the number of enlarged loops (over four times the normal afferent, transition, and efferent limbs width), and the number of giant capillary loops (10 or more times the normal width of capillary limbs). The vascular deletion score was assessed according to Lee and colleagues' method [23], in which a deletion area is defined as the absence of two or more consecutive loops. Each finger was rated from 0 to 3: 0 = no deletion area; 1 = one or two discrete deletion areas; 2 = more than two discrete deletion areas; 3 = extensive and confluent deletion areas. For each patient, the NFC parameters were calculated as the average obtained in all analyzed digits. All NFCs were performed by the same observer, blinded for the patient conditions.

### Fingertip skin blood flow before and after cold stimulus

The blood flow of the dorsum of four fingertips (excluding the thumb) of the left hand was measured using a laser Doppler imager (Moor LDI-VR; Moor Instruments, Axminster, UK) before and after CS. All individuals were in a sitting position with the left arm resting at heart level. The laser Doppler imager uses a red helium-neon laser operating at 633 nm with a penetration of approximately 1 mm depth in the skin that is directed onto the area of interest by a computer-controlled mirror. All images were obtained at scan speeds of 4 ms/pixel with a time of acquirement of 3 minutes and 15 seconds for each image. The distance between the photodetector and the examined surface was 40 cm, yielding an examined area of 168.5 cm^2 ^(10.4 cm × 16.2 cm). The blood flow of the dorsum of the four fingertips was determined by establishing four regions of interests at each fingertip, defined as an area from the proximal interphalangeal joint up to and including the nailbed. The global mean finger blood flow (FBF) of the four fingertips was derived (Moor LDI system software V5.2) and averaged. Blood flow was displayed in arbitrary perfusion units (PU). The variability of FBF between the four different fingers was also evaluated.

After baseline blood flow measurement, patients underwent a CS (submersion of both hands in water at 15°C for 1 minute) (UNITEMP 116; Fanem, São Paulo, SP, Brazil). Further laser Doppler scanning was performed along a 30-minute interval after CS. Blood flow measurements were made at 1 minute, 4 minutes and 15 seconds, 10 minutes and 45 seconds, 17 minutes and 15 seconds, 20 minutes and 30 seconds, and at 27 minutes after CS.

Reproducibility of LDI was evaluated for four SSc patients and four controls who underwent the procedure on two different occasions 4 weeks apart. The basal mean FBF was evaluated.

### Cold stimulus fingertip lacticemy test

FTL was determined on the fourth left finger before (pre-CS-FTL) and after (post-CS-FTL) CS. After the baseline LDI measurement, a brisk puncture was performed at the volar surface of the fingertip with an automatic device (Softclix; Boehringer-Mannheim, Germany) and the first blood drop was adsorbed onto a lactate strip (Softclix, Boehringer-Mannheim, Germany). The strip was processed immediately in a portable spectrophotometer (Accusport, Boehringer-Mannheim, Germany). FTL was determined again 10 minutes after CS (post-CS-FTL).

### Statistical analysis

All results are expressed as the mean ± standard deviation. Differences among groups were analyzed using Student's *t *test or the Mann-Whitney test. Longitudinal comparison among successive time points was analyzed by repeated-measures analysis of variance. Spearman's correlation coefficient was used to correlate FBF, FTL, and NFC parameters. Reproducibility of LDI was evaluated by Pearson's correlation coefficient. *P *< 0.05 was considered significant. Statistical analysis was performed using SPSS statistical software (version 15 SPSSInc.,Chicago,IL,USA).

## Results

Forty-four SSc patients (three male, 41 female; 47.4 ± 10.5 years old) and 40 healthy controls (three male, 37 female; 46.9 ± 8.1 years old) were included with no statistical difference in age or gender between the two groups (Table 1). Disease duration was 5.9 ± 5.5 years. Seven patients (15.9%) had early SSc according to the LeRoy and Medger criteria [22], 19 patients (43.1%) had limited cutaneous SSc, and 18 patients (40.9%) had diffuse cutaneous SSc. Twenty patients were using calcium channel blockers, five patients were using captopril (11.3%), and three patients were using losartan (6.8%).

**Table 1:** Demographic, clinical and laboratory data of patients with systemic sclerosis and healthy controls

	Systemic sclerosis patients (*n *= 44)	Healthy controls (*n *= 40)	*P *value
Age (years)	47.4 ± 10.5	46.9 ± 8.1	0.807
Gender (female/male)	41/3	37/3	0.904
Duration of Raynaud's phenomenon (years)	8.0 ± 7.1	-	-
Disease duration (years)	5.9 ± 5.5	-	-
Presence of digital pitting scars, fingertip resorption, or digit amputation	25 (56.8%)	-	-
Antinuclear antibody	37 (84.1%)	-	-
Anti-Scl70 antibody	3 (6.8%)	-	-
Anti-centromere antibody	13 (29.5%)	-	-
Number of patients using calcium channel blockers (%)	20 (45%)	-	-

Baseline FBF was significantly lower in SSc patients as compared with controls (296.9 ± 208.8 vs. 503.6 ± 146.4 PU, respectively; *P *< 0.001). The same was true at all time points after CS (*P *< 0.001) (Table 2).

**Table 2:** Finger blood flow before and after cold stimulus in controls and in systemic sclerosis patients

	Finger blood flow (perfusion units)		
		
	Systemic sclerosis patients	Healthy controls	*P *value
Baseline FBF	296.9 ± 208.8	503.6 ± 146.4	<0.001
T1 FBF	113.5 ± 86.0	290.2 ± 131.0	<0.001
T4 FBF	155.1 ± 155.6	361.6 ± 177.0	<0.001
T10 FBF	221.3 ± 198.4	412.4 ± 172.1	<0.001
T17 FBF	218.3 ± 196.5	380.8 ± 147.2	<0.001
T20 FBF	224.3 ± 198.7	387.2 ± 142.9	<0.001
T27 FBF	209.9 ± 185.2	437.7 ± 171.8	<0.001

Data presented as mean ± standard deviation. Mean finger blood flow (FBF) at baseline and after 15°C cold stimulus at different time points: T1, 1 minute; T4, 4 minutes and 15 seconds; T10, 10 minutes and 45 seconds; T17, 17 minutes and 15 seconds; T20, 20 minutes and 30 seconds; and T27, 27 minutes after cold stimulus.

**Figure 1:**
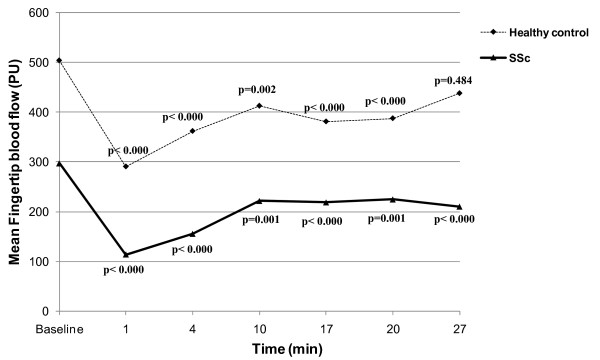
**Mean finger blood flow at baseline and after cold stimulus**. Mean finger blood flow (FBF) at baseline and after cold stimulus (CS) in healthy controls and in systemic sclerosis (SSc) patients. *P *values correspond to the comparison between mean FBF at different time points after CS in relation to the baseline FBF. PU, perfusion units.

There was a significant decrease in FBF after CS as compared with baseline in SSc patients and in controls, followed by recovering of FBF 27 minutes after CS in healthy controls (*P *= 0.484) but not in SSc patients (*P *< 0.000) (Figure 1).

There was no correlation between age or disease duration and baseline FBF (*r *= 0.011, *P *= 0.944 and *r *= -0.237, *P *= 0.121, respectively). Interestingly, the baseline FBF values tended to be lower in the group of 25 patients with digital scars and previous ulceration/amputation in comparison with the group of 19 patients with absence of these abnormalities (218.35 ± 147.53 vs. 336.38 ± 223.5 PU, respectively; *P *= 0.06).

There was no difference in the FBF values in the 18 patients with diffuse cutaneous SSc as compared with the 26 patients with limited cutaneous or early SSc at baseline (286.5 ± 188.8 vs. 304.0 ± 225.0 PU, respectively; *P *= 0.924) or after CS (data not shown).

The mean coefficient of variation of basal FBF between the four fingers evaluated was 30% in SSc patients (range, 3.4 to 54%), and 10.9% in controls (range, 3.03 to 26.4%). Reproducibility of LDI showed an intraclass correlation coefficient of 0.980 (*P *< 0.001), with 95% limits of agreement of -49.86 to 27.74.

**Figure 2:**
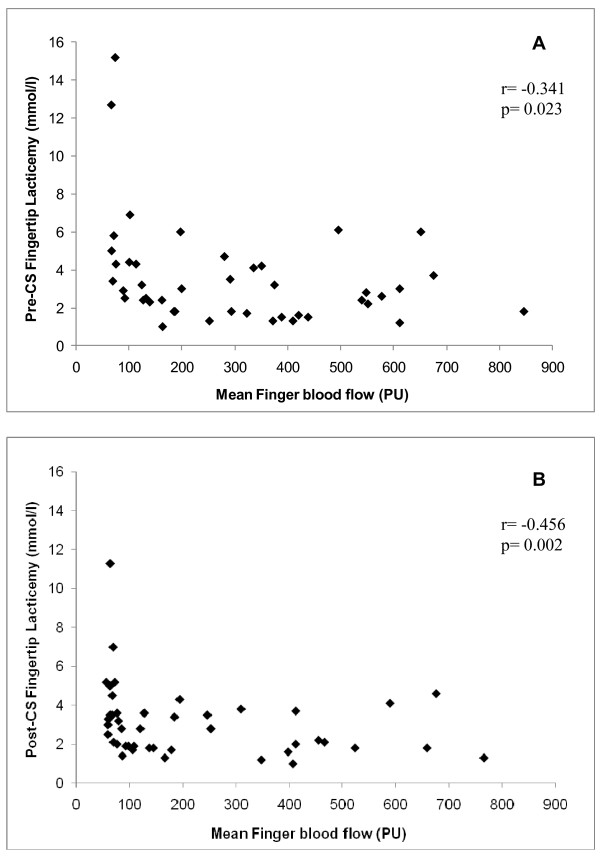
**Correlation between fingertip lacticemy and mean finger blood flow**. Correlation between fingertip lacticemy and mean finger blood flow (FBF) **(a) **before and **(b) **10 minutes after cold stimulus (CS) in systemic sclerosis (SSc) patients and healthy controls. PU, perfusion units.

The CS-FTL test showed higher pre-CS-FTL and post-CS-FTL values in patients with SSc than in normal controls (pre-CS-FTL, 3.5 ± 2.7 vs. 2.4 ± 0.8 mg/dl, respectively; *P *= 0.04; and post-CS-FTL, 2.7 ± 1.8 vs. 1.6 ± 0.7 mg/dl, respectively; *P *< 0.001). There was a considerable decrease in FTL 10 minutes after CS in normal controls (Percentage difference between post- and pre-CS-FTL = -30.4 ± 20.3%) and only a slight decrease in SSc patients (Percentage difference between post- and pre-CS-FTL = -8.2 ± 43.5%) (*P *= 0.02). Interestingly, there was a negative correlation between FTL and FBF at baseline (*r *= -0.341, *P *= 0.023) and 10 minutes after CS (*r *= -0.456, *P *= 0.002) (Figure 2).

As expected, NFC showed a lower number of capillary loops per millimeter in SSc patients than in controls (*P *< 0.001), as well as a higher number of enlarged capillary loops (*P *< 0.001), giant capillary loops (*P *< 0.001), and higher vascular deletion scores (*P *< 0.001) (Table 3). There was no correlation between baseline FBF and the NFC parameters in SSc patients (number of capillary loops/mm, *r *= 0.091, *P *= 0.56; number of enlarged capillary loops, *r *= -0.025, *P *= 0.87; number of giant capillary loops, *r *= 0.046, *P *= 0.76; vascular deletion score, *r *= -0.289, *P *= 0.22).

**Table 3:** Nailfold capillaroscopy parameters in systemic sclerosis patients and healthy controls

	Nailfold capillaroscopy parameter			
	
	Number of capillary loops/mm	Number of enlarged capillary loops	Number of giant capillary loops	Vascular deletion score
Healthy controls	10.1 ± 0.6	0.1 ± 0.2	0.0 ± 0.0	0.0 ± 0.0
SSc patients	6.7 ± 1.9	4.5 ± 2.7	0.3 ± 0.6	1.5 ± 0.9
*P *value^a^	<0.001	<0.001	<0.001	<0.001

^a^Healthy controls vs. systemic sclerosis (SSc) patients.

## Discussion

The technique of LDI is currently considered a promising method for the study of microvascular involvement in systemic rheumatic diseases [24]. The present study originally evaluated the peripheral microcirculation using LDI in association with two other methods that address different aspects of SSc microangiopathy. The present study therefore integrated parameters of microvascular morphological abnormalities, assessed by NFC, and the dynamic behavior of microvascular blood flow before and after a vasoreactive stimulus, assessed by LDI and FTL. SSc patients showed decreased digital perfusion, as demonstrated by lower FBF and higher FTL at baseline and after CS in comparison with healthy controls. In addition, SSc patients exhibited a delay in digital blood flow recovery after CS, when compared with healthy controls, as demonstrated by LDI. There was a moderate negative correlation between FBF and FTL at baseline and after CS, indicating that SSc patients with a more severe decrease in FBF, as measured by LDI, presented a higher degree of tissue hypoxia. SSc patients presented also the typical NFC scleroderma pattern characterized by enlarged and giant capillaries, and loss of capillaries. There was no correlation, however, between functional and morphological microvascular abnormalities measured by LDI and NFC, suggesting that these are complementary tools for evaluation of SSc microangiopathy.

Interestingly, FBF tended to be lower in patients with digital scars and previous ulcers or amputation - suggesting that LDI could provide information on the severity of peripheral vascular involvement in SSc. This finding, however, deserves further investigation with a larger sample of patients.

Several techniques have been developed to study microcirculation function and for the measurement of microvascular blood flow in patients with primary RP or secondary RP - such as NFC, thermographic imaging, plethysmography, single-point laser Doppler flowmetry, and LDI [5,25]. The laser Doppler technique has proven high sensitivity for measuring changes in digital skin perfusion in response to a dynamic challenge [26]. Although single-point laser Doppler flowmetry has been used in many studies to quantify blood flow in patients with RP, the experience with LDI is still limited. LDI allows the evaluation of larger areas of the microcirculation as compared with other techniques, and provides a direct measurement of blood flow in a given area of the skin, as opposed to indirect methods such as thermography.

The findings of lower baseline FBF and higher FTL in SSc patients when compared with healthy controls are supported by previous reports of severe narrowing of capillaries and arterioles at the fingertips, devascularization, and decreased blood flow in the microcirculation of these patients [3,27], and are consistent with the concept that the deranged arteriolar/capillary bed in this disease ensues a chronic hypoxic regimen in the fingertips. Cold-induced changes have been used to provide an evaluation of the degree of vasospasm in SSc patients and to obtain information on the prognosis and management of these patients [17,20,28]. The absence of FBF recovery until 30 minutes after CS observed in the present report is consistent with several studies that showed a higher reactivity after cold exposure and a delayed blood flow recovery in SSc [20,29]. An array of disturbances in blood flow dynamics observed in SSc results in a slow and less effective capillary blood flow after CS. The SSc microangiopathy involves dramatic dysfunction in vascular regulatory mechanisms that presents initially with neural abnormalities, increased release of endothelin-1, and reduced release of nitric oxide and prostacyclin, and progresses to structural derangement of the involved vessels [3,30]. As a result of proliferation and fibrosis of the intimal layer, vessels cannot compensate the critical impairment in blood flow during RP attacks [31].

The present findings are also in agreement with previous studies using LDI for the evaluation of peripheral blood flow in SSc patients. Picart and colleagues used LDI before, during, and after CS (cold pad at 5°C for 8 minutes) in eight SSc patients, 10 patients with primary RP, and seven healthy controls [20]. As in our study, SSc patients showed significantly lower perfusion before, during, and after CS as compared with controls. Murray and colleagues evaluated 16 patients with SSc, 14 patients with primary RP, and 16 healthy controls using LDI before and after the same CS used in our study [17]. The authors observed lower baseline perfusion and a lower rewarming/reperfusion curve in patients with SSc compared with healthy controls. Finally, Seifalian and colleagues [32] and Rosato and colleagues [33] showed lower baseline mean blood flow in the hands of SSc patients compared with healthy controls at room temperature, but no cold challenge was applied.

An interesting point in the present study was the integration of functional parameters (LDI and FTL) and morphological features of SSc microangiopathy. The CS-FTL test previously developed by Pucinelli and colleagues in 2002 has demonstrated consistent abnormalities before and after CS in SSc patients [13], characterized by higher FTL values in comparison with primary RP and healthy controls. The data reported herein confirm our previous findings of higher FTL in resting conditions and after CS in patients with SSc [14,15]. These findings correlated nicely with the lower baseline perfusion and longer recovery time observed with the LDI method. This correlation is also consistent with the concept of an association between decreased blood flow and tissue hypoxia in patients with SSc.

The structural abnormalities of the microcirculation were evaluated by NFC, the gold standard assessment method for structural microvascular damage in systemic rheumatic diseases [33]. Our findings confirmed that NFC is a sensitive method for identification of SSc microangiopathy [17]. The absence of correlation between the microvascular morphological abnormalities depicted by NFC and the digital blood flow evaluated by LDI in SSc is not surprising since the two methods evaluate different aspects of the microcirculation. NFC evaluates morphological abnormalities specifically on the capillaries of the nailfold bed, which are relatively stable and usually do not change significantly during short intervals of follow-up [34]. In the LDI system, the type of vessels evaluated depends on the penetration and absorption of the laser wavelength. The red laser light (633 nm) used in the present study has penetration depth of 0.5 to 1 mm in excised skin. This light is more likely to be back-scattered to the detector from larger, thermoregulatory-type microvessels, having passed through the capillaries without significant interaction [16]. Additionally, since cutaneous microcirculation is a dynamic system with functions such as thermoregulation and metabolism, many environmental (temperature) and individual factors (hormones, stress, vasoconstrictors medications) may influence the cutaneous blood flow measured by LDI [35]. Murray and colleagues showed only a weak negative correlation between the intercapillary distance measured by NFC under 300× magnification and the initial gradient in the first 2 minutes after CS measured by LDI [17]. They could not find any other correlation between other nailfold capillary parameters and LDI.

## Conclusions

The finding of a decreased FBF at baseline and after CS in patients with SSc indicates that LDI allows sensitive and objective measurement of the digital skin blood flow in patients with RP and SSc. Additionally, the inverse correlation between FTL and FBF reveals the deleterious consequences of decreased blood flow on tissues, since high FTL reflects tissue hypoxia. Finally, the lack of correlation between functional and morphological microvascular abnormalities measured by LDI and NFC suggests that it is appropriate to consider LDI and NFC as complementary tools for evaluation of different aspects of SSc microangiopathy. Further studies evaluating LDI as a technique for monitoring treatment response and prospective studies with long follow-up are warranted to investigate a possible role for LDI in the evaluation of disease extension and severity of SSc.

## Abbreviations

CS: cold stimulus; FBF: finger blood flow; FTL: fingertip lacticemy; LDI: laser Doppler imaging; NFC: nailfold capillaroscopy; PU: perfusion units; RP: Raynaud's phenomenon; SSc: systemic sclerosis.

## Competing interests

The authors declare that they have no competing interests.

## Authors' contributions

MJUC and CK participated in the study design, acquisition of data, analysis and interpretation of data, and manuscript preparation. LECA participated in the study design, contributed to analysis and interpretation of data, and assisted in drafting of the manuscript. All authors approved the final manuscript.
